# *PASmiR*: a literature-curated database for miRNA molecular regulation in plant response to abiotic stress

**DOI:** 10.1186/1471-2229-13-33

**Published:** 2013-03-01

**Authors:** Shihua Zhang, Yi Yue, Liang Sheng, Yunzhi Wu, Guohua Fan, Ao Li, Xiaoyi Hu, Mingzhu ShangGuan, Chaoling Wei

**Affiliations:** 1 School of life sciences, Anhui Agricultural University, Hefei 230036, China; 2 Key laboratory of Tea Biochemistry and Biotechnology, Ministry of Education, Ministry of Agriculture, Anhui Agricultural University, Hefei 230036, China; 3 College of information and computer science, Anhui Agricultural University, Hefei 230036, China; 4 Department of Electronic Science and Technology, University of Science and Technology of China, Hefei, China

## Abstract

**Background:**

Over 200 published studies of more than 30 plant species have reported a role for miRNAs in regulating responses to abiotic stresses. However, data from these individual reports has not been collected into a single database. The lack of a curated database of stress-related miRNAs limits research in this field, and thus a cohesive database system should necessarily be constructed for data deposit and further application.

**Description:**

*PASmiR*, a literature-curated and web-accessible database, was developed to provide detailed, searchable descriptions of miRNA molecular regulation in different plant abiotic stresses. *PASmiR* currently includes data from ~200 published studies, representing 1038 regulatory relationships between 682 miRNAs and 35 abiotic stresses in 33 plant species. *PASmiR*’s interface allows users to retrieve miRNA-stress regulatory entries by keyword search using plant species, abiotic stress, and miRNA identifier. Each entry upon keyword query contains detailed regulation information for a specific miRNA, including species name, miRNA identifier, stress name, miRNA expression pattern, detection method for miRNA expression, a reference literature, and target gene(s) of the miRNA extracted from the corresponding reference or miRBase. Users can also contribute novel regulatory entries by using a web-based submission page. The *PASmiR* database is freely accessible from the two URLs of http://hi.ustc.edu.cn:8080/PASmiR, and http://pcsb.ahau.edu.cn:8080/PASmiR.

**Conclusion:**

The *PASmiR* database provides a solid platform for collection, standardization, and searching of miRNA-abiotic stress regulation data in plants. As such this database will be a comprehensive repository for miRNA regulatory mechanisms involved in plant response to abiotic stresses for the plant stress physiology community.

## Background

MicroRNAs (miRNAs) are endogenous, single-stranded, small (~21-23nt), non-coding, regulatory RNA molecules that manipulate messenger RNA (mRNA) degradation and translation repression by binding complementary sites in the protein-encoding, or 3’-untranslated regions, of target mRNAs [1-3]. Contemporary research has revealed numerous miRNAs in diverse plant species, such as *Arabidopsis thaliana* (mouse-ear cress), *Oryza sativa* (Asian rice), *Chlamydomonas reinhardtii* (flagellated green algae), and *Populus euphratica* (subtropical poplar tree) [4-7]. An abundance of recent evidence suggests that plant miRNAs are critical to several essential biological processes in plant physiology, such as floral organ identity, leaf morphogenesis, cellular signaling, and stress responses [8-11]. Because of the diverse and important functions of miRNAs in plants, the dysfunction, mutation, and dysregulation of specific miRNAs has the potential to negatively influence plant growth and productivity.

Initially discovered as regulators of developmental timing in *Caenorbabditis elegans* (transparent nematode), miRNAs have already been demonstrated to play important regulatory roles, particularly in the complex network of gene regulatory pathways. During abiotic stress to plants, such as drought, salinity, wounding, and high temperature, miRNAs act at the posttranscriptional level in gene regulatory networks associated with stress adaptation and tolerance. Under adverse stress conditions, gene expression can be elaborately regulated to facilitate the action of these tolerance mechanisms [12-14], providing further evidence of the importance of miRNAs in regulation of plant responses to abiotic stress conditions.

In the past decade, the regulatory mechanisms of miRNAs involved in plant stress responses have garnered increasing attention. For example, miRNAs have been identified to regulate plant response to drought [15,16], salinity [17,18], low temperature [19,20], and nutrient deficiency [21-23]. More recently, a set of conserved and non-conserved miRNAs from *Medicago truncatula* (barrel clover) [24], *Oryza sativa* (Asian rice) [25], *Brassica napus* (rapeseed) [26], and *Arabidopsis thaliana* (mouse-ear cress) [27] that act to regulate plant response to heavy metal exposure, have been idenfified. These findings indicated that certain stress conditions stimulate specific plant species to produce miRNAs involved in species-specific regulatory processes that vary highly between different species, potentially accounting for differences in plant tolerances to specific stress conditions. Additionally, these results indicate that miRNAs could represent a discriminative evolutionary footprint associated with abiotic stress regulation, relationships that can only be determined by comprehensive organization of miRNA regulation data from many plant species.

To date, more than one thousand miRNAs have been discovered in diverse plant species [28]. Many of these miRNAs have confirmed involvement in regulatory networks associated with plant stress response. Despite notable recent progress in identifying miRNA regulation during stress conditions, detailed data of miRNA molecular regulation in different plant abiotic stresses, such as miRNA expression pattern and target genes of miRNAs, remains widely distributed in published literature. In the current stage, a cohesive database system is sorely needed for data deposit and further application. Therefore, development of the literature-curated database system “*PASmiR*” (*miR*NA molecular regulation in *P*lant *A*biotic *S*tress) for the storage of regulatory relationships between miRNAs and plant abiotic stresses will be a valuable resource for contemporary researchers. While these immediate, tangible benefits are apparent, the *PASmiR* database may also serve as a valuable ongoing program for the study of miRNA regulatory mechanisms associated with plant responses to abiotic stress as additional information becomes available in this field.

## Construction and content

### Collection of regulatory relationships between miRNA and plant abiotic stress

We searched the databases of the PubMed, Scopus, Google Scholar, and Ovid [29-32], by using keywords, such as ‘stress microRNA’ and ‘stress miRNA’, to retrieve publications that described miRNA-stress regulatory relationships. *PASmiR* currently encompasses data from approximately 200 published studies, representing 1038 regulatory relationships between 682 miRNAs and 35 abiotic stresses in 33 plant species. For each of these regulatory relationships, we collected detailed information for miRNA molecular regulation in abiotic stresse response, which contains the plant species name, a miRNA identifier, the abiotic stress name, miRNA expression pattern (up-regulated or down-regulated) during stress condition, detection method for miRNA expression (e.g., miRNA microarray, qRT-PCR, northern blot, and deep sequencing, etc.), a reference literature, the target gene(s) of miRNA extracted from the corresponding reference, and validated (predicted) target gene(s) derived from miRBase [33]. In *PASmiR*, convenient links are provided to other external databases, including miRNA sequence and annotation in miRBase, and species taxonomic description from UniProt taxonomy [34]. In the original publications, different members of a miRNA family may not be stated properly due to limited experimental design. Thus, a family-like miRNA identifier (e.g., osa-miR172) documented with expression pattern assignment will denote the regulatory pattern of an unspecified family member.

### Nomenclature standardization

The published studies used to create *PASmiR* entries use varied standards for miRNA and abiotic stress presentation, differing widely between individual journals and researchers. As a result, variant miRNA and abiotic stress spellings and descriptions exist, making literature curation a complicated task. For example, two forms of osa-miR171 and miR-171 that appear in different publications may actually refer to the same miRNA in Oryza sativa (Asian rice) [19,35].

Establishment of the *PASmiR* database necessitated the development of a curated standard nomenclature for miRNAs and abiotic stresses. Each miRNA inputted into the *PASmiR* database was converted to a standard terminology by referring to miRBase [36], as follows: a prefix of species abbreviation based on Latin name was followed by a dash, "miR", and a family number. For instance, “osa-miR172” would be correct terminology for *Oryza sativa* miRNA from family 172.

**Figure 1:**
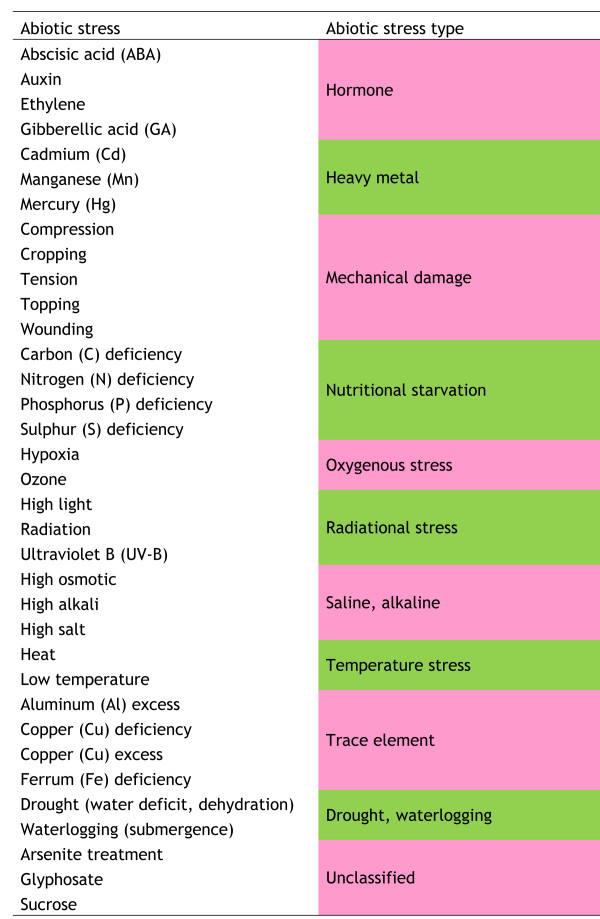
Eleven abiotic stress classes generated by manual curation.

**Figure 2:**
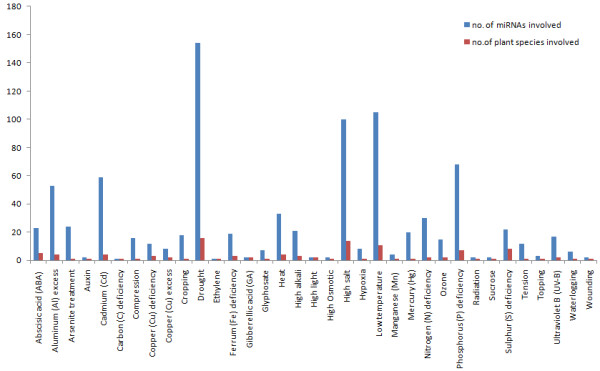
**Distribution of miRNAs and plant species involved in 35 different abiotic stresses.** Examination of the prevalent abiotic stresses represented in *PASmiR* demonstrated miRNA involvement was most often reported in stresses of drought, low temperature, high salt, and phosphate (Pi) deficiency.

A total of 35 different plant abiotic stresses have been identified. For the purposes of inclusion in *PASmiR*, each inputted abiotic stress was assigned unique names and manually classified into one of 11 abiotic stress classes according to physiological affection (Figure 1). Figure 2 illustrates the distribution of miRNAs and plant species involved in different abiotic stresses. Examination of the prevalent abiotic stresses represented in *PASmiR* demonstrated miRNA involvement was most often reported in stresses of drought, low temperature, high salt, and phosphate (Pi) deficiency.

### Database construction

The *PASmiR* database was constructed using freely available and open source frameworks, such as Apache, JavaServer Pages (JSP), MySQL, Struts2, Spring, Hibernate and so on. The curated miRNA-stress regulatory relationships were stored in a MySQL Server and accessed using Structured Query Language (SQL) queries. The web platform is based on Apache Tomcat server and its pages are generated via a combination of Java language and JSP scripts. In addition, Hibernate modules were applied to manipulate data and convert data formats.

## Utility

### Keyword-based searching

**Figure 3:**
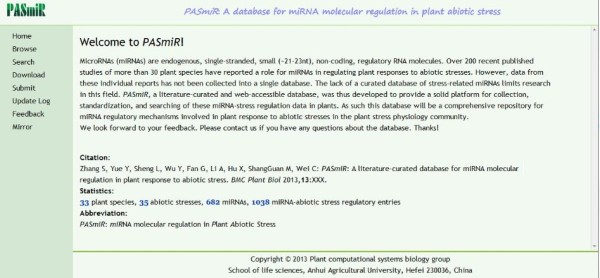
**Homepage of the *PASmiR *database.** The web-accessible *PASmiR* database allows for curated miRNA-stress regulatory entries to be clearly and simply browsed, searched, downloaded, and updated.

The web-accessible *PASmiR* database allows for curated miRNA-stress regulatory relationships to be clearly and simply browsed, searched, downloaded, and updated (Figure 3). Using the search page, remote users can search the database by keywords. Three search fields are present that may be used together or separately: 1) plant species, 2) abiotic stress, and 3) miRNA. Logical operators (AND and OR) were configured between two of these fields to allow rapid, targeted access to specific entries of interest.

### Fuzzy search capabilities

Users can remotely access the web-based user interface to conveniently generate queries on the database using logical, standardized keywords. *PASmiR* also offers a fuzzy search engine, allowing querying of miRNA-stress regulatory information by plant species, abiotic stress or miRNA, even when the exact name is unknown. Notably, multiple hits may result from input of single queries containing few or vague keywords, allowing for manual selection of data presented by relevance to the search term(s).

### User submit to PASmiR

*PASmiR* provides a submit page that allows non-affiliated researchers to independently contribute novel miRNA-stress regulatory relationships as they become available. For user submissions, primary required fields are: 1) plant species, 2) abiotic stress, 3) miRNA and 4) reference title, as indicated by the conventional red star symbol. The curator(s) at our site conducts manual verification of the original publication(s) for data validation purposes to maintain the quality and integrity of the database. Submissions that pass this review process are then approved for entry into the *PASmiR* database. Notably, all new submission will be made available in coming *PASmiR* versions on a monthly release schedule.

## Discussion

Contemporary exploration of miRNA molecular regulation associated with plant abiotic stresses has demonstrated that miRNAs act as important regulators in plant physiological adaption mechanisms during response to unfavor-able stresses. Unfortunately, data information on specific miRNAs involved in regulation of plant responses to abiotic stress is widely dispersed throughout current scientific literature. To provide a comprehensive repository for collection, standardization, and searching of these miRNA-abiotic stress regulatory relationships, the literature-curated and web-accessible *PASmiR* database was established.

Notably, the number of miRNAs involved in particular abiotic stress responses is highly dependent on the experimental approaches proposed in the original investigations. For example, more miRNAs will be observed using a microarray platform than other low-throughput technologies [37,38]. In different plant species, miRNAs may be defined as functionally conserved if their homologs behave as regulators of specific abiotic stress responses in more than one other plant species. For example, the miRNA homologs peu-miR164 and mtr-miR164 are functionally conserved in plant responses to drought stress in both *Populus euphratica* (Euphrates Poplar) [6] and *Medicago truncatula* (Barrel Clover) [39]. Of 682 total plant miRNAs, 242 (35.5%) represent functional conserved miRNAs, and 16 (6.6%) of which are functionally conserved in more than 3 abiotic stresses response in different plant species (see Additional file 1). Investigation of these conserved miRNAs using a structured database approach, such as that provided by *PASmiR,* will facilitate improvements in the understanding of stress-focused miRNA functional evolutionarily relationship in plant species. This data has potential applications for experimental biology aiming to improve plant crop performance and breeding characteristics.

**Figure 4:**
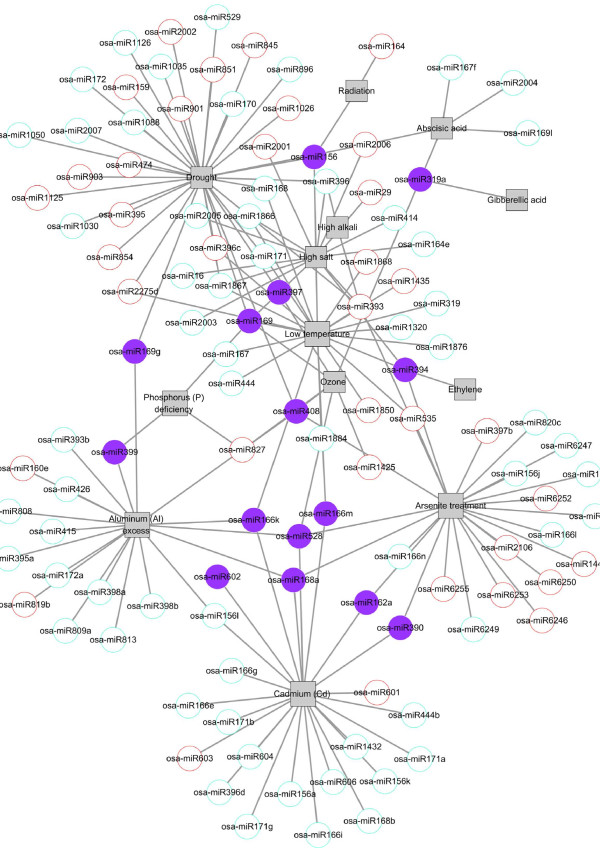
**Bipartite network visualization of the regulatory relationship between miRNAs and abiotic stresses in the commercial crop *Oryza sativa *(Asian rice).** In the network, squares and circles correspond to stresses and miRNAs, respectively. A link was placed between stresses and miRNAs indicating miRNA involvement in the regulation of a specific abiotic stress response. The outlined red, outlined blue, and solid purple of the circle denote different miRNA expression pattern (up-regulated, down-regulated and combined, respectively) in different stress conditions, respectively. Notably, a family-like miRNA identifier (e.g., osa-miR172) with expression pattern assignment will denote the regulatory pattern of an unspecified family member.

Current efforts have demonstrated that miRNAs play critical roles in regulating plant responses to abiotic stresses in a complicated network manner [40-43]. Thus, it is possible that analysis of the miRNA-stress regulation data archived in *PASmiR* may be able to yield novel information about the network regulatory mechanisms of miRNAs involved in stress response. In the staple food crop *Oryza sativa* (Asian rice), a bipartite network can be used to describe the 162 regulatory relationships between 113 miRNAs and 13 abiotic stresses known in this species (Figure 4). Within the network, drought, arsenite treatment, cadmium (Cd), low temperature, aluminum (Al) excess, and high salt demonstrate the highest connectivity by involving 35, 25, 25, 22, 19, and 13 regulatory miRNAs in the stress response, respectively.

Several miRNAs, such as osa-miR156, osa-miR393, and osa-miR528, have been demonstrated to play regulatory roles in more than two types of abiotic stress responses. Among these miRNAs, a small portion (solid purple circles) exhibit opposite expression patterns in different abiotic stresses response. The miRNA osa-miR156, for example, has a high-expression tendency when subjected to radiation exposure, but exhibits the opposite tendency when exposed to either low temperature or drought, indicating that, as a regulator of stress, osa-miR156 exhibits unique behaviors under variant stress conditions. Cumulatively, these findings indicate the complex network regulatory relationships between miRNAs and abiotic stresses. By using *PASmiR*, researchers will have access to a comprehensive knowledge base for assembling bipartite networks for plant species documented, allowing the completion of novel derivations for detailed stress-driven miRNA molecular regulatory mechanisms.

The *PASmiR* database project, reported herein, provides the initial groundwork for an ongoing program for cataloging and utilizing miRNA-abiotic stress regulatory information in plants. Notably, the current version is not as comprehensive as we envision future versions to be. Ongoing collection and curation of new publications related to miRNA-stress response regulation is essential to this program’s success. New versions of the databases are currently scheduled to be released on a monthly basis, incorporating all new submissions to the database over the preceding month. While this work is currently time and resource intensive, the pending employment of a natural language processing (NLP) technique to allow more rapid abstract prescreening will facilitate the addition of many new entries to the database in the near future. This strategy is expected to increase comprehensiveness and accessibility of the database, promoting broader interest from researchers worldwide.

## Conclusion

We constructed the first comprehensive database for miRNA molecular regulation in plant abiotic stress responses. The web-based program was created by manually reviewing widely scattered scientific literature. This database, however, also provides a convenient submission interface for contribution of novel miRNA-stress regulatory relationship by independent researchers. *PASmiR* provides comprehensive, keyword-searchable miRNA-abiotic regulatory relationships focused on a specific plant species, abiotic stress, and miRNA family. Thus, *PASmiR* will aid in rapid and complete exploration of the complex network of miRNA regulatory mechanisms involved in plant abiotic stress responses, serving as a valuable resource for future investigators in experimental biology concerned with commercial crop breeding and characteristics.

## Availability and requirements

Project name: *PASmiR*: A literature-curated database for miRNA molecular regulation in plant response to abiotic stress

Project home pages: http://hi.ustc.edu.cn:8080/PASmiR

http://pcsb.ahau.edu.cn:8080/PASmiR

Operating system(s): Platform independent

Programming languages: JSP, MySQL, HTML and JavaScript

License: Not required.

Any restrictions to use by non-academics: None

## Abbreviations

PASmiR: *miR*NA molecular regulation in *P*lant *A*biotic *S*tress; microRNA: miRNA.

## Competing interests

The authors declare that they have no competing interests.

## Authors' contributions

SZ, YY and LS performed literature mining, constructed the database platform, and wrote the manuscript. YW, GF, AL, XH and MS helped with the design of database platform and update of the database, and provided scientific suggestions and criticisms for improving the manuscript and website. SZ and CW participated in the design, helped write the manuscript and supervised the whole project. All authors read and approved the final manuscript.

## Supplementary Material

Additional file 1 Distribution of functionally conserved miRNAs in 35 different abitotic stresses. The expression patterns of miRNAs are indicated as red and blue colors according to their up-regulated and down-regulated patterns, respectively. Click here for file
